# RIS-Enabled Secret Key Generation for Secured Vehicular Communication in the Presence of Denial-of-Service Attacks

**DOI:** 10.3390/s23084104

**Published:** 2023-04-19

**Authors:** Mahmoud A. Shawky, Syed Tariq Shah, Qammer H. Abbasi, Mohamed Hussein, Muhammad A. Imran, Syed Faraz Hasan, Shuja Ansari, Ahmad Taha

**Affiliations:** 1 James Watt School of Engineering, University of Glasgow, Glasgow G12 8QQ, UK; syedtariq.shah@glasgow.ac.uk (S.T.S.); qammer.abbasi@glasgow.ac.uk (Q.H.A.); muhammad.imran@glasgow.ac.uk (M.A.I.); ahmad.taha@glasgow.ac.uk (A.T.); 2 Department of Communications Engineering, Military Technical College, Cairo 11646, Egypt; mhusse5@mtc.edu.eg; 3 Directorate of Research Services, University of New England, Armidale, NSW 2351, Australia; faraz.hasan@une.edu.au

**Keywords:** Chebyshev chaotic mapping, denial-of-service attacks, physical-layer security, reconfigurable intelligent surfaces, secret key extraction

## Abstract

Wireless communication has become an integral part of modern vehicles. However, securing the information exchanged between interconnected terminals poses a significant challenge. Effective security solutions should be computationally inexpensive, ultra-reliable, and capable of operating in any wireless propagation environment. Physical layer secret key generation has emerged as a promising technique, which leverages the inherent randomness of wireless-channel responses in amplitude and phase to generate high-entropy symmetric shared keys. The sensitivity of the channel-phase responses to the distance between network terminals makes this technique a viable solution for secure vehicular communication, given the dynamic behavior of these terminals. However, the practical implementation of this technique in vehicular communication is hindered by fluctuations in the communication link between line-of-sight (LoS) and non-line-of-sight (NLoS) conditions. This study introduces a key-generation approach that uses a reconfigurable intelligent surface (RIS) to secure message exchange in vehicular communication. The RIS improves the performance of key extraction in scenarios with low signal-to-noise ratios (SNRs) and NLoS conditions. Additionally, it enhances the network’s security against denial-of-service (DoS) attacks. In this context, we propose an efficient RIS configuration optimization technique that reinforces the signals received from legitimate users and weakens the signals from potential adversaries. The effectiveness of the proposed scheme is evaluated through practical implementation using a 1-bit RIS with 64×64 elements and software-defined radios operating within the 5G frequency band. The results demonstrate improved key-extraction performance and increased resistance to DoS attacks. The hardware implementation of the proposed approach further validated its effectiveness in enhancing key-extraction performance in terms of the key generation and mismatch rates, while reducing the effect of the DoS attacks on the network.

## 1. Introduction

The integration of wireless technology into modern vehicles has greatly enhanced the exchange of crucial information such as location, speed, and direction, offering drivers real-time traffic updates and reducing the risk of accidents [1]. However, the wireless channel used for this exchange can also be vulnerable to malicious attacks, such as the interception and alteration of transmitted data [2,3]. Public-key cryptography (PKC) has been widely used to secure these communications and protect privacy. However, PKC-based key management faces power-consumption and computational-complexity challenges [4]. Symmetric-key cryptography is a more effective alternative in that respect. However, establishing a symmetric shared key using cryptographic operations still depends on PKC-based approaches such as Diffie–Hellman key exchange [5].

In this context, the study of secret key generation through the physical-layer security mechanism has gained increasing attention, especially since Maurer proposed the idea in 1993 [6]. The unique properties of wireless channels, such as their time-varying, short-term reciprocity, and space–time uniqueness within the coherence interval $T_{c}$, provide a natural source for generating secret keys [7]. The key-generation process involves channel probing, measurement quantization, information reconciliation, and privacy amplification, resulting in the establishment of a secure shared key between the sender and receiver [8]. The secret key capacity is a crucial aspect of this field of study. Theoretical upper limits of secret key capacity have been established through the works of Ahlswede and Csiszar et al. [9] in source-type and channel-type models with wiretappers. Subsequently, research on the secret key capacity under more complex system models has been developed. The channel-phase response is a valuable resource for secure key extraction, as it is highly sensitive to the distance between terminals [10]. This makes it effective in highly dynamic vehicular networks, generating high-entropy cryptographic keys. However, it is important to consider the shadowing effect from surrounding terminals (i.e., vehicles and infrastructures) that can lead to unpredictable channel-fading variations in urban and rural areas. Additionally, the key-extraction performance is adversely affected by the decrease in signal-to-noise ratio (SNR) values. This limitation results in reduced PHY-layer key-extraction performance for non-line-of-sight (NLoS) communication scenarios and long-range applications.

Recently, the reconfigurable intelligent surface (RIS) field has emerged as a technology that can improve communication quality by adjusting reflection coefficients such as phase, amplitude, frequency, or polarization [11]. The RIS has been employed in various applications, such as satellite communications [12], physical-layer security [13], and internet-of-things (IoT) networks [14], demonstrating its versatility and the potential for diverse use cases. RISs comprise many passive reflecting units that can be placed at adaptable locations and independently alter the incident signal, therefore improving signal transmission [15]. In recent years, some researchers have applied RISs to the physical-layer security of wireless communications to improve the secrecy data rate under the wiretap channel, a concept referred to as keyless information theory security [16]. However, the application of RISs to phase-based key-extraction mechanisms has not yet been explored. The effective extraction of keys with the aid of the RIS and the use of channel-phase responses to generate shared keys remains an open issue in the field of physical-layer security technology. Given the ability of RISs to configure the wireless channel in real time through passive reflection, they have the potential to improve secret key capacity significantly. Furthermore, flooding attacks are a potential hazard, whereby the attacker floods the network with a substantial volume of simultaneous communication requests, thus constituting a Denial-of-Service (DoS) attack [17]. By strengthening the signal from a certain side (i.e., a legitimate user) while weakening it from another (i.e., the adversary), the RIS can help mitigate the effect of such attacks. An effective way to accomplish this is to configure the RIS elements in a way that can destructively interfere in one direction, and constructively interfere in another.

In summary, the main contributions of this work are as follows:

- This study extends our previous work introduced in [5] by proposing a RIS-assisted key-extraction method that enhances the signal strength for the designated user’s location while reducing that from the active attacker’s location. Hence, the proposed method improves the key-extraction performance for designated users while mitigating the impact of DoS attacks within the network.
- To accomplish this, a RIS configuration optimization algorithm is designed using the Hadamard matrix codebook. This algorithm optimizes the measurement quantization order based on the optimal configuration’s average SNR estimate.
- The proposed RIS optimization algorithm is practically implemented using a 1-bit RIS with 64×64 elements and two Universal Serial Radio Peripherals (USRPs) operating in the 5G communication frequency range (3.75 GHz). Finally, the statistical randomness of the extracted keys is measured to demonstrate the extracted key suitability for use as cryptographic keys.

The structure of this paper is as follows: Section 2 provides an overview of the existing phase-based key-extraction method. Section 3 presents the preliminary concepts required for this research. Section 4 presents the proposed RIS-assisted key-extraction method. Section 5 analyses the hardware implementation of the method. Finally, Section 6 summarizes the findings and contributions of this work.

## 2. Related Works

In the channel-probing step, imperfect channel reciprocity results in a mismatching error in the extracted bits [18]. To address this issue, the authors in [19] employed the difference in the phase estimates between two signals of different frequencies sinusoids as a randomness source to reduce the channel non-reciprocity component impact, and consequently the mismatching probability. Likewise, the study in [20] improved extraction performance using the phase differentials and amplitudes as distinct sources of randomness. This approach leads to an improvement in the key-generation rate and subsequently accelerates the process of symmetric-key establishment. In [21], a round-trip group key-generation mechanism is proposed. This mechanism entails a member of a group of nodes initiating two signals with random phases and transmitting them through a ring group of nodes in clockwise and counterclockwise directions. By combining the channel response estimates generated by each node from both directions, a high degree of correlation is achieved, enabling the generation of a shared group key. However, while the mechanism demonstrates theoretical efficacy, its practical implementation is hindered by the accumulation of noise across multiple group nodes. Furthermore, the requirement for the entire channel-probing process between all nodes to be completed within $T_{c}$ poses a challenge. This time frame is often too short, given the short coherence period of high-speed terminals.

Although all these studies carefully design the quantization process, quantized observations near the boundary regions can result in a high rate of mismatches. To overcome this limitation, the work in [22] explored the use of guard intervals to reduce the probability of mismatches. However, a trade-off exists between the mismatching probability and the bit extraction rate. Larger boundary regions decrease the mismatching probability and result in a lower bit extraction rate due to the greater number of dropped observations. Multicarrier communication systems encompass partitioning the frequency spectrum into a multitude of parallel subcarriers, which are then used as independent sources of randomness. In [10], a single-side probing mechanism was proposed for an orthogonal frequency division multiplexing (OFDM) system consisting of *N* subcarriers. The mechanism involves initiating random phase sequences by a terminal and using the reciprocal characteristics of the channel to mask the mapped preliminary shared key.

In the quantization step of the measurement process, optimizing the size of the quantization region is critical for optimizing the performance of the extraction process [23]. The authors in [24] employed the channel gain complement method to counteract the impact of hardware imperfections, such as the carrier frequency offset. It optimizes the thresholding regions of the multi-level quantization process. The mentioned works are premised on a crucial assumption: that all nodes within the network are separated by a minimum distance of (λ/2) to ensure that the channel responses between network terminals are decorrelated within $T_{c}$. In other words, it is important to ensure that all the network terminals are geographically apart by a distance of at least λ/2, ensuring that their estimates are decorrelated within $T_{c}$. This requirement can be practically ensured for vehicle-to-vehicle (V2V) communication through proper physical security measures. However, ensuring such physical security in vehicle-to-infrastructure (V2I) communication may be difficult. An attacker could potentially gain access to the secret features of surrounding terminals by stacking readily available cards onto the transceivers of roadside units. To solve the previously discussed challenge, we present a Diffie–Hellman channel-probing mechanism, detailed in [5], incorporating the Chebyshev chaotic mapping function. Implementing this mechanism negates the need for a minimum (λ/2) distance between network terminals, therefore enhancing the efficiency of V2I applications. However, a key challenge in the performance of key extraction remains the dependence of its performance on the SNR value of the received signal. In certain scenarios, such as shadowing or long-distance communication, the SNR can be very low, presenting a significant obstacle in the effective extraction of keys. This highlights the need for further research to address this issue and develop practical solutions to enhance key-extraction performance under such conditions. This paper aims to assess the efficacy of using the RIS in improving key-extraction performance while operating under the effect of DoS attacks in which a large volume of traffic or data is sent to a targeted system, overwhelming its resources and causing it to become unavailable to legitimate users [25]. It is worth mentioning that this type of attack is significant for high-complexity cryptographic-based key-agreement techniques.

## 3. Preliminaries

This section provides a brief overview of the secret key extraction process [5]. A thorough discussion of our considered system model is also provided. The notations used in this paper are summarized in Table 1 for better readability.

### 3.1. Review of the PHY-Layer Secret Key Extraction Scheme in [5]

**Figure 1:**
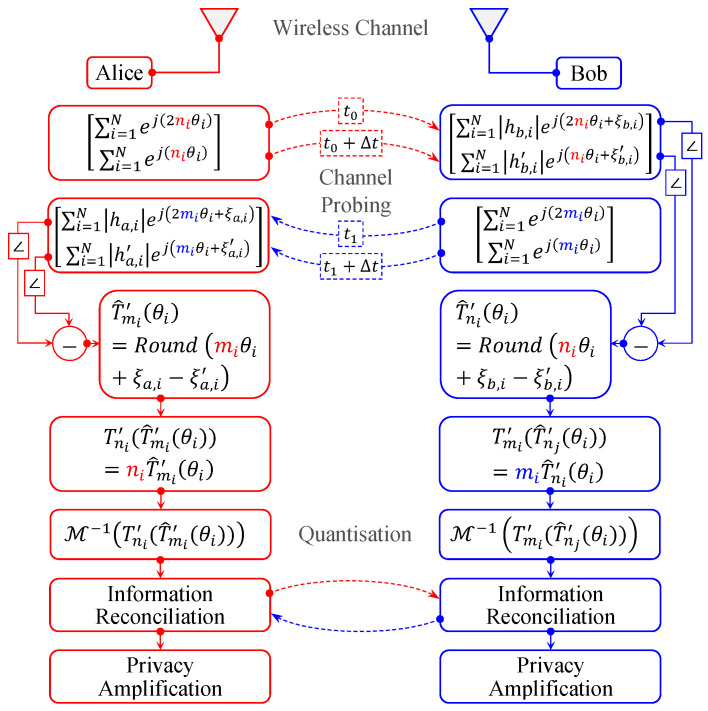
The PHY-layer secret key extraction scheme in a noiseless channel.

The work introduced in [5] proposes a novel Diffie–Hellman channel-probing mechanism that uses the extended Chebyshev chaotic mapping operation to exchange probing signals in an interleaved fashion. Specifically, the extended Chebyshev mapping operation for the OFDM system of *N* subcarriers is formulated as

(1) $$T_{n_{i}}^{\prime }\left(\theta_{i}\right)=\left\{\begin{array}{cc} n_{i}\cdot \theta_{i}\;\mathrm{mod}\;p, & \theta_{i}\in \left[0,2\pi \right)\\ n_{i}\cdot \cos^{-1}\left(x_{i}\right)\;\mathrm{mod}\;p, & x_{i}=\cos\left(\theta_{i}\right) \end{array}\right.\;\mathrm{for}\;i=1,\ldots ,N,$$

where p=2π, $n_{i}$ is a large integer number, and $\theta_{i}=\frac{2\Pi }{2^{r}}$ for r∈{1,2,3} is the primitive root of the *i*th subcarrier. The primitive root $\theta_{i}$ is a generator of the group G such that its multiples generate the entire group. For example, let r=2, then $\theta_{i}=\frac{\Pi }{2}$. Thus, the cyclic group elements are $G_{2}=\left\{0,\frac{\Pi }{2},\Pi ,\frac{3\Pi }{2}\right\}$. For r=3, $\theta_{i}=\frac{\Pi }{4}$. Thus, the cyclic group elements are $G_{3}=\left\{0,\frac{\Pi }{4},\frac{\Pi }{2},\frac{3\Pi }{4},\Pi ,\frac{5\Pi }{4},\frac{3\Pi }{2},\frac{7\Pi }{4}\right\}$. We have considered a scenario where two parties (Alice and Bob) are in the same communication range and want to establish a secure communication link. In this context, Alice and Bob exchange authenticated probing packets at times $t_{0}$ and $t_{1}$, respectively. Based on the received probing packets, both terminals can extract a high-entropy secret key, which is used to secure subsequent transmissions using the upper layer’s crypto-based approaches. Figure 1 reviews the steps involved in the secret key extraction process in [5]. Generally, the extraction process comprises channel probing and quantization, information reconciliation, and privacy amplification. In the former, Alice sends the probing packet in the form of two OFDM symbols of *N* subcarriers, which can be represented in a simplified form as:

(2) $$\begin{array}{cc} \; & s_{a}\left(t_{0}\right)=\sum_{i=1}^{N}\sqrt{\frac{2E_{S}}{T}}e^{j\left(T_{2n_{i}}^{\prime }\left(\theta_{i}\right)\right)}=\sum_{i=1}^{N}\sqrt{\frac{2E_{S}}{T}}e^{j\left(2n_{i}\theta_{i}\right)}\\ \; & s_{a}\left(t_{0}+\Delta t\right)=\sum_{i=1}^{N}\sqrt{\frac{2E_{S}}{T}}e^{j\left(T_{n_{i}}^{\prime }\left(\theta_{i}\right)\right)}=\sum_{i=1}^{N}\sqrt{\frac{2E_{s}}{T}}e^{j\left(n_{i}\theta_{i}\right)}, \end{array}$$

where the transmission time interval between both OFDM symbols is $\Delta t\leq T_{c}$. Thus, Bob’s received signal can be expressed as

(3) $$\begin{array}{c} r_{b}\left(t_{0}^{\prime }\right)=\sum_{i=1}^{N}\sqrt{\frac{2{\left|h_{i}\right|}^{2}E_{s}}{T}}e^{j\left(T_{2n_{i}}^{\prime }\left(\theta_{i}\right)+\xi_{b,i}\right)}+N_{i}\\ r_{b}\left(t_{0}^{\prime }+\Delta t\right)=\sum_{i=1}^{N}\sqrt{\frac{2{\left|h_{i}^{\prime }\right|}^{2}E_{s}}{T}}e^{j\left(T_{n_{i}}^{\prime }\left(\theta_{i}\right)+\xi_{b,i}^{\prime }\right)+}N_{i}^{\prime }, \end{array}$$

where $\left\{|h_{i}|,|h_{i}^{\prime }|\right\}$ and $\left\{\xi_{i},\xi_{i}^{\prime }\right\}$ are the channel-fading coefficients and phase responses of the *i*th subcarrier at times $\left\{t_{0}^{\prime },t_{0}^{\prime }+\Delta t\right\}$, respectively and $\left\{N_{i},N_{i}^{\prime }\right\}$ are complex additive Gaussian noises $\mathrm{CN}\left(0,\sigma_{n}^{2}\right)$ with zero means and $\sigma_{n}^{2}$ variances. It is noteworthy to mention that the channel responses $\left\{|h_{i}|,\xi_{i}\right\}$ are highly correlated with $\left\{|h_{i}^{\prime }|,\xi_{i}^{\prime }\right\}$ for $\Delta t\leq T_{c}$. Similarly, Bob replies by sending an authenticated probing packet as in (2) with phases $\left\{T_{2m_{i}}^{\prime }\left(\theta_{i}\right),T_{m_{i}}^{\prime }\left(\theta_{i}\right)\right\}$ at times $\left\{t_{1}^{\prime },t_{1}^{\prime }+\Delta t\right\}$. Then, both terminals, Alice and Bob, equalize their received signals by computing $e_{a}\left(t\right)=r_{a}\left(t_{1}^{\prime }\right)r_{a}{\left(t_{1}^{\prime }+\Delta t\right)}^{*}$ and $e_{b}\left(t\right)=r_{b}\left(t_{0}^{\prime }\right)r_{b}{\left(t_{0}^{\prime }+\Delta t\right)}^{*}$, respectively. Hence, the phases of $e_{a}\left(t\right)$ and $e_{b}\left(t\right)$ of the *i*th subcarrier can be formulated as

(4) $$\begin{array}{cc} \; & ∠e_{a,i}\left(t\right)=m_{i}\theta_{i}+\left(\xi_{a,i}-\xi_{a,i}^{\prime }\right)+\left(\omega_{a,i}-\omega_{a,i}^{\prime }\right)\\ \; & ∠e_{b,i}\left(t\right)=n_{i}\theta_{i}+\left(\xi_{b,i}-\xi_{b,i}^{\prime }\right)+\left(\omega_{b,i}-\omega_{b,i}^{\prime }\right), \end{array}$$

where $\left\{\omega_{a,i},\omega_{a,i}^{\prime }\right\}$ and $\left\{\omega_{b,i},\omega_{b,i}^{\prime }\right\}$ are the noisy added estimates result from $\left\{N_{i},N_{i}^{\prime }\right\}$ in (3) at the sides of Alice and Bob, respectively with Gaussian distributions $N\left(0,\sigma^{2}\right)$. Accordingly, both terminals use the Round function to obtain ${\hat{T}}_{m_{i}}^{\prime }\left(\theta_{i}\right)$ and ${\hat{T}}_{n_{i}}^{\prime }\left(\theta_{i}\right)$ as

(5) $$\begin{array}{ccc} \; & {\hat{T}}_{m_{i}}^{\prime }\left(\theta_{i}\right) & =Round\left(∠e_{a,i}\left(t\right)\right)=Round\left(m_{i}\theta_{i}+\left(\xi_{a,i}-\xi_{a,i}^{\prime }\right)+\left(\omega_{a,i}-\omega_{a,i}^{\prime }\right)\right)\\ \; & {\hat{T}}_{n_{i}}^{\prime }\left(\theta_{i}\right) & =Round\left(∠e_{b,i}\left(t\right)\right)=Round\left(n_{i}\theta_{i}+\left(\xi_{b,i}-\xi_{b,i}^{\prime }\right)+\left(\omega_{b,i}-\omega_{b,i}^{\prime }\right)\right), \end{array}$$

where the function Round(x) is used to round *x* to the nearest multiple of $2\pi /2^{r}$ for r∈{1,2,3}. Then, Alice and Bob compute $T_{n_{i}m_{i}}^{\prime }\left(\theta_{i}\right){|}_{Alice}=T_{n_{i}}^{\prime }\left({\hat{T}}_{m_{i}}^{\prime }\left(\theta_{i}\right)\right)$ and $T_{n_{i}m_{i}}^{\prime }\left(\theta_{i}\right){|}_{Bob}=T_{m_{i}}^{\prime }\left({\hat{T}}_{n_{i}}^{\prime }\left(\theta_{i}\right)\right)$, respectively. The use of the Round function in the context is important to avoid the significant error results from multiplying the negligible value of $\left(\left(\xi -\xi^{\prime }\right)+\left(\omega -\omega^{\prime }\right)\right)$ by the large integer number $n_{i}$ or $m_{i}$. Finally, both terminals quantize their estimates to convert them into bit streams using a mapping operation $M^{-1}\left(.\right)$ of order *r*. For clarity, a Gray code mapping operation of order 2 can be expressed as

(6) $$M^{-1}\left(T_{n_{i}m_{i}}^{\prime }\left(\theta_{i}\right)\right)=\left\{\begin{array}{cc} 00 & T_{n_{i}m_{i}}^{\prime }\left(\theta_{i}\right)\in \left[-\frac{\pi }{4},\frac{\pi }{4}\right)\\ 01 & T_{n_{i}m_{i}}^{\prime }\left(\theta_{i}\right)\in \left[\frac{\pi }{4},\frac{3\pi }{4}\right)\\ 11 & T_{n_{i}m_{i}}^{\prime }\left(\theta_{i}\right)\in \left[\frac{3\pi }{4},-\frac{3\pi }{4}\right)\\ 10 & T_{n_{i}m_{i}}^{\prime }\left(\theta_{i}\right)\in \left[-\frac{3\pi }{4},-\frac{\pi }{4}\right) \end{array}\right.\;\mathrm{for}\;i=1,\ldots ,N.$$

Note that the higher the variance $\sigma^{2}$ of the phase noisy estimates in (4), the lower the quantization order *r*, and vice versa [5].

### 3.2. System Model

**Figure 2:**
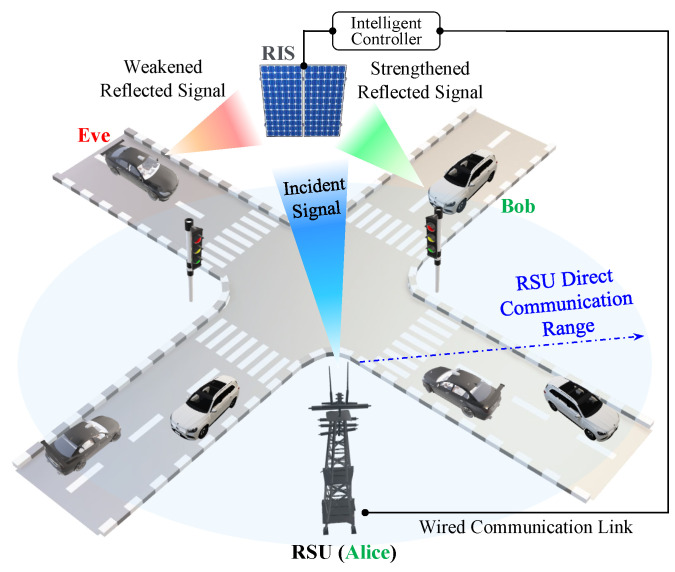
System modeling.

In this study, the vehicular communication network comprises the following entities, as shown in Figure 2.

1. The RSU: RSUs are stationary devices located along roads that facilitate wireless communication between themselves and surrounding vehicles within a particular range. Each RSU acts as a relay between vehicles, extending the communication range and improving the network’s reliability. It is equipped with wireless communication capabilities and can support various applications, such as traffic management, safety warnings, and entertainment services. It also has a reliable communication link with the RIS’s intelligent controller, so configurations of reflecting units can be optimized. Through this mechanism, the RSU effectively manages the RIS to enhance the transmission of signals towards a designated direction while simultaneously reducing the strength of signals toward potential unauthorized interceptors, commonly referred to as “Eve”.
2. The vehicle’s onboard units (OBUs): OBU is a communication device installed within each vehicle in the network. It can communicate with other OBUs and RSUs within range, facilitating the exchange of traffic-related messages in 100–300 ms intervals based on the dedicated short-range communication protocol [26]. In this way, OBUs play a crucial role in the functioning of the vehicular network.
3. The RIS: RISs are intelligent surfaces that can dynamically change their electromagnetic behaviors to improve the performance of wireless networks. RISs can be used to manipulate the propagation of radio signals, allowing for better signal quality, increased network coverage, and improved energy efficiency. The intelligent controller is an integral component of each RIS. It manages and configures the multiple meta-surface reflecting units (RUs) of order *N* elements that make up the RIS. It plays a crucial role in optimizing the performance of the RIS in the network.
4. The adversary Eve: “Eve” is an active attacker who overloads the network with excessive traffic, causing it to become unavailable to legitimate users. In this attack, the adversary overwhelms the target’s resources and prevents it from functioning properly, therefore denying service to its intended users. By constructing and launching a flooding DoS attack, the attacker aims to disrupt the system’s normal functioning and cause inconvenience or harm to its users.

## 4. RIS-Assisted Secret Key Extraction Method

This section shows how the RIS improves the key-extraction performance and reduces the impact of potential flooding-based DoS attacks on the network.

### 4.1. Performance Optimization

Three critical evaluation metrics must be considered while optimizing the key-extraction performance, namely the bit generation rate (BGR), the bit mismatch rate (BMR), and the secret bit generation rate (SBGR). The BGR is a measure of the efficiency of this process and typically represents the number of generated bits per channel sample, expressed as:

(7) $$BGR=\frac{\;\mathrm{Total}\;\mathrm{extracted}\;\mathrm{bits}\;}{\;\mathrm{Channel}\;\mathrm{sample}\;}.$$

A high valuation of the BGR indicates a more efficient extraction process and a higher rate of secret bit generation, resulting in improved security and faster key establishment for the communication system. On the other hand, the BMR represents the number of mismatched bits extracted from each channel sample, expressed as:

(8) $$BMR=\frac{\;\mathrm{No}.\;\mathrm{of}\;\mathrm{mismatched}\;\mathrm{bits}\;}{\;\mathrm{Channel}\;\mathrm{sample}\;}.$$

We define the SBGR as the number of matched bits, which is represented as SBGR=BGR−BMR. Hence, the SBGR considers both the BGR and the BMR in the process of secret key extraction. For negligible channel-phase decorrelation $\left(\xi -\xi^{\prime }\right)\approx 0$, the phase distribution of the equalized signal ∠e(t) in (4) is normally distributed with means $\left\{T_{n_{i}}^{\prime }\left(\theta_{i}\right)=n_{i}\theta_{i},T_{m_{i}}^{\prime }\left(\theta_{i}\right)=m_{i}\theta_{i}\right\}$ and variance $2\sigma^{2}$ for {Alice, Bob}, respectively [5]. Thus, its cumulative distribution function (CDF) is approximated as:

(9) $$\begin{array}{c} \phi \left(x\right)=\frac{1}{2}\left[1+\mathrm{erf}\left(\frac{x-T_{n_{i}\left(m_{i}\right)}^{\prime }\left(\theta_{i}\right)}{2\sigma }\right)\right],\\ \mathrm{erf}\left(z\right)=\frac{2}{\sqrt{\pi }}\int_{0}^{z}e^{-t^{2}}dt \end{array}$$

where erf(z) is the error function. Thus, the probability of error $P_{e}$ is the probability of the estimated ∠e(t) in (4) to be out of the interval $\left[T_{n_{i}\left(m_{i}\right)}^{\prime }+\frac{\pi }{2^{r}},T_{n_{i}\left(m_{i}\right)}^{\prime }-\frac{\pi }{2^{r}}\right)$, which can be represented by:

(10) $$P_{e}=2\phi \left(T_{n_{i}\left(m_{i}\right)}^{\prime }\left(\theta_{i}\right)-\frac{\pi }{2^{r}}\right).$$

Accordingly, the communicating terminals can agree on the optimum quantization order r∈{1,2,3} for an acceptable $P_{e}\leq a_{1}$ as:

(11) $$x=\arg\max_{x^{\prime }}\mathrm{erf}\left(\frac{x^{\prime }-T_{n_{i}\left(m_{i}\right)}^{\prime }\left(\theta_{i}\right)}{2\sigma }\right)\leq a_{1}-1.$$

Based on *x*, *r* is optimized as:

(12) $$r=\arg\max_{r^{\prime }}2^{r^{\prime }}\leq \frac{\pi }{x}\;\;\mathrm{for}\;r^{\prime }=1,2,3.$$

### 4.2. Channel Modeling

**Figure 3:**
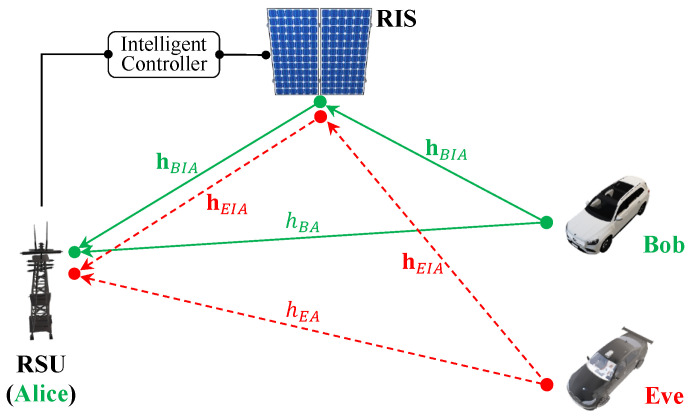
RIS-assisted channel modeling.

The scenario depicted in Figure 3 involves the concurrent processes of communication establishment between Bob and Alice, and Eve’s deliberate disruption of network integrity through the inundation of the network with excessive communication requests. In this scenario, the RSU can manage the RIS and optimize its configuration to reinforce the signal in the direction of the intended recipient “Bob”, while simultaneously mitigating the strength of the signals received from the adversary “Eve”. Hence, the signals received by Alice from both Bob and Eve can be theoretically formulated as follows:

(13) $$\begin{array}{cc} y_{A}|Bob & =\left(h_{BA}+h_{BIA}\right)x+N_{A}\\ \; & =\left(h_{BA}+\sum_{i=1}^{N}h_{BIA}^{i}\beta_{i}\Psi_{i}\right)x+N_{A}\\ y_{A}|Eve & =\left(h_{EA}+h_{EIA}\right)x+N_{A}\\ \; & =\left(h_{EA}+\sum_{i=1}^{N}h_{EIA}^{i}\beta_{i}\Psi_{i}\right)x+N_{A}, \end{array}$$

where $N_{A}$ is the complex additive Gaussian noise $CN\left(0,\sigma_{n}^{2}\right)$, $\left\{h_{BA},h_{EA}\right\}$ are the channel responses in the complex form of the direct link from (Bob→Alice) and (Eve→Alice), respectively, and $\left\{h_{BIA},h_{EIA}\right\}$ are the superposition of the *N* channel multipath components of the RIS’s elements of the indirect link from (Bob→RIS→Alice) and (Eve→RIS→Alice), respectively. Additionally, the configuration of the RIS is represented by the variable $H={\left[\beta_{1}\Psi_{1},\beta_{2}\Psi_{2},\ldots ,\beta_{N}\Psi_{N}\right]}^{T}$, where $\left\{\beta_{i},\Psi_{i}\right\}$ defines the state of each RIS element. An example of a 1-bit RIS can be described as follows: the phase shift applied by each unit cell, denoted by $\Psi_{i}$, is equal to Π, and the reflection coefficient, represented by $\beta_{i}$, is a binary variable that can take on values of either 0 or 1.

The use of the RIS helps increase the secret key capacity *I*, which refers to the maximum amount of information that can be securely extracted from the physical layer of a communication system and used as a secret key. By properly designing and controlling the phase shifts applied by the RIS, the RIS can counter the effects of fading and interference in the channel, which can also result in higher secret key capacities. Therefore, the RIS can be seen as a valuable tool for improving the secret key capacity *I* in the presence of an eavesdropper and ensuring secure communication. The work in [16] provides a theoretical formulation for the secret key capacity denoted by:

(14) $$I=\log_{2}\left(1+\frac{{\left(\sigma_{h_{BA}}^{2}+\sum_{i=1}^{N}\beta_{i}^{2}\sigma_{h_{BIA}^{i}}^{2}\right)}^{2}/\sigma_{n}^{4}}{1+2\left(\sigma_{h_{BA}}^{2}+\sum_{i=1}^{N}\beta_{i}^{2}\sigma_{h_{BIA}^{i}}^{2}\right)/\sigma_{n}^{2}}\right).$$

The RIS can adjust signal directionality, consequently reducing the signal strength from Eve’s direction and enhancing the signal coming from Bob. This can be achieved by adjusting the phase shifts applied by each unit cell of the RIS so that the reflection coefficients of the unit cells constructively interfere in certain directions and destructively interfere in others. Therefore, the goal is to optimize the RIS configuration *H* to maximize the secret key capacity *I* in (14) while concurrently reducing any interference from Eve.

### 4.3. Optimizing the Best RIS Configuration $\left(H_{opt}\right)$

The use of the Hadamard matrix in the configuration of the RIS offers several advantages, including low complexity, high efficiency, and improved performance. This makes the Hadamard matrix effective for scenarios where reducing interference, enhancing privacy, and increasing energy efficiency are critical objectives in wireless communication systems [27]. The Hadamard matrix offers a suite of orthogonal and binary phase shift values that can be applied to the elements of the RIS to influence the reflection of incoming electromagnetic waves in a specific direction or with a preferred phase shift. The flexibility and efficacy of the Hadamard matrix in configuring the RIS to achieve these objectives while minimizing complexity makes it a promising solution for wireless communication challenges. This paper involves the measurement of the average signal-to-noise ratio ($\bar{\mathrm{SNR}}$) for every configuration (*H*) of the OFDM system. Based on these measurements, we developed an optimization method for the RIS configuration, which is presented in Algorithm 1. This method encompasses four phases: initialization, scanning toward Bob, scanning toward Eve, and configuration optimization.

- Initialization: Alice initializes the Hadamard codebook $HD=\sum_{i=1}^{N_{x}\times N_{y}}H_{i}$, where $N_{x}$ and $N_{y}$ are the number of elements in the RIS’s *x* and *y* coordinates, respectively.
- Scanning toward Bob: Alice scans the average SNR value for the received OFDM symbols from Bob, denoted as ${\bar{\mathrm{SNR}}}_{i}^{Bob}$, for each configuration $H_{i}$ within the set of all possible configurations, HD, where $i=1,2,\ldots ,N_{x}\times N_{y}$.
- Scanning toward Eve: Alice scans the average SNR value of the received OFDM symbols from Eve, denoted as ${\bar{\mathrm{SNR}}}_{i}^{Eve}$, for each configuration $H_{i}$ within the set of all possible configurations, HD, where $i=1,2,\ldots ,N_{x}\times N_{y}$.
- Configuration optimization: Alice computes the ratio of the average SNR for Bob ${\bar{\mathrm{SNR}}}_{i}^{Bob}$ over the average SNR for Eve ${\bar{\mathrm{SNR}}}_{i}^{Eve}$, denoted as $C_{i}$, for $i=1,2,\ldots ,N_{x}\times N_{y}$. The maximum value of $C_{i}$, referred to as $C_{max}$, is then determined from the set of all values of $C_{i}$. The optimum configuration, denoted as $H_{opt}$, is identified as the configuration $H_{i}$ that corresponds to the maximum value of $C_{max}$. This calculation maximizes Bob’s average SNR while minimizing Eve’s average SNR.

**Algorithm 1** Optimizing the Best RIS Configuration $\left(H_{opt}\right)$.Initialization1:The Hadamard codebook $HD=\sum_{i=1}^{N_{x}\times N_{y}}H_{i}$ for the $\left(N_{x}\times N_{y}\right)$ RIS reflecting units2:Two empty variables, $SNR^{Bob}$ and $SNR^{Eve}$, used to store the measured SNRs3:An empty variable *C*Alice is communicating with the legitimate terminal (Bob)4:**for** 
$i=1:(N_{x}\times N_{y})$
**do**5:      Measuring the average SNR value $\left({\bar{\mathrm{SNR}}}_{i}^{Bob}\right)$ for each Hadamard matrix $\left(H_{i}\right)$6:      Appending the measured ${\bar{\mathrm{SNR}}}_{i}^{Bob}$ to $SNR^{Bob}$7:
**end for**
Alice is communicating with the illegitimate terminal (Eve)8:**for** 
$i=1:(N_{x}\times N_{y})$
**do**9:      Measuring the average SNR value $\left({\bar{\mathrm{SNR}}}_{i}^{Eve}\right)$ for each Hadamard matrix $\left(H_{i}\right)$10:      Appending the measured ${\bar{\mathrm{SNR}}}_{i}^{Eve}$ to $SNR^{Eve}$11:
**end for**
Optimizing the best configuration12:**for** 
$i=1:(N_{x}\times N_{y})$
**do**13:      Computing $C_{i}=\frac{{\bar{\mathrm{SNR}}}_{i}^{Bob}}{{\bar{\mathrm{SNR}}}_{i}^{Eve}}$14:      Appending the computed $C_{i}$ to *C*15:
**end for**
16:Finding the best configuration ($H_{opt}=H_{i}$) corresponding to $C_{max}=\max\left(C_{i}\in C\right)$

## 5. Hardware Implementation Analysis

In this section, we present the hardware-based experimental results for the proposed RIS-assisted secret key-extraction method, and evaluate the effectiveness of the proposed optimization approach for configuring the RIS.

### 5.1. Experimental Setup and the RIS Configuration Analysis

**Figure 4:**
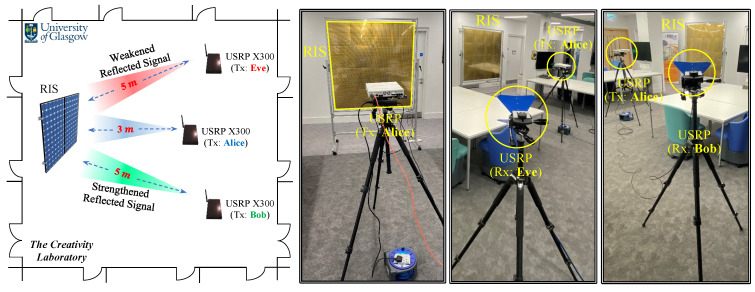
Experiment setup for the secret key generation scheme.

We describe the experimental parameters in the following before evaluating the proposed method. As depicted in Figure 4, the experimental setup consists of two universal serial radio peripherals (USRPs) version Ettus X300 and a 1-bit RIS with 64×64 elements. One USRP serves as the transmitter, positioned 3 m from the RIS, while the other USRP is equipped with two channels with horn antennas and serves as two separate receivers, representing Bob and Eve, positioned 5 m from the RIS and situated at $45^{\circ }$ degrees on either side of the line connecting the RIS and the first USRP. In this experiment, a single antenna is installed on all terminals. The carrier frequency is set to 3.75 GHz, and the sampling rate is configured at 200 KHz for an OFDM system with 256 subcarriers.

**Figure 5:**
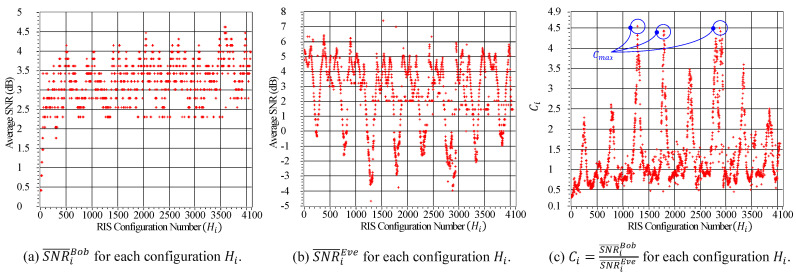
The average SNR values for different configurations and their optimized value.

We calculated ${\bar{\mathrm{SNR}}}_{i}^{Bob}$ and ${\bar{\mathrm{SNR}}}_{i}^{Eve}$ for each configuration matrix $H_{i}\in HD$, where HD is the Hadamard codebook of order |HD|=64×64=4096 configurations. Figure 5a illustrates the relationship between ${\bar{\mathrm{SNR}}}_{i}^{Bob}$ and $H_{i}$, while Figure 5b presents the relationship between ${\bar{\mathrm{SNR}}}_{i}^{Eve}$ and $H_{i}$, for i=1,…,4096. It can be observed from Figure 5b that some configurations enhance the transmitted signals’ received power, while others result in a reduction ranging from −3.5 dB to 6.5 dB. We applied Algorithm 1 to the estimated measurements to compute $C_{i}=\frac{{\bar{\mathrm{SNR}}}_{i}^{Bob}}{{\bar{\mathrm{SNR}}}_{i}^{Eve}}$ for each configuration, as shown in Figure 5c. This figure shows that the configurations associated with the top three peaks are good candidates for $H_{opt}$. Therefore, we maximize the value of $C_{i}$ to determine $H_{opt}$.

**Figure 6:**
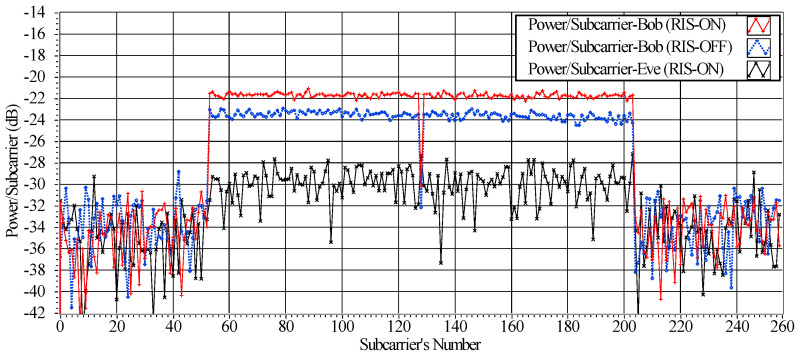
The power/subcarrier for N=256 at the side of Bob and Eve, with/without the RIS.

In Figure 6, we display the impact of the RIS on the received OFDM symbols at the sides of Bob and Eve. When the RIS is activated using the optimized configuration $H_{opt}$, it is evident that the received power at Bob’s side is boosted by approximately 2 dB compared to the scenario when the RIS is turned off. Additionally, the figure highlights the effectiveness of the RIS in reducing the received power at Eve’s side. This reduced received power at Eve’s side effectively reduces the impact of DoS attacks carried out by Eve.

### 5.2. Implementation Results and Analysis of the Key-Extraction Process

**Figure 7:**
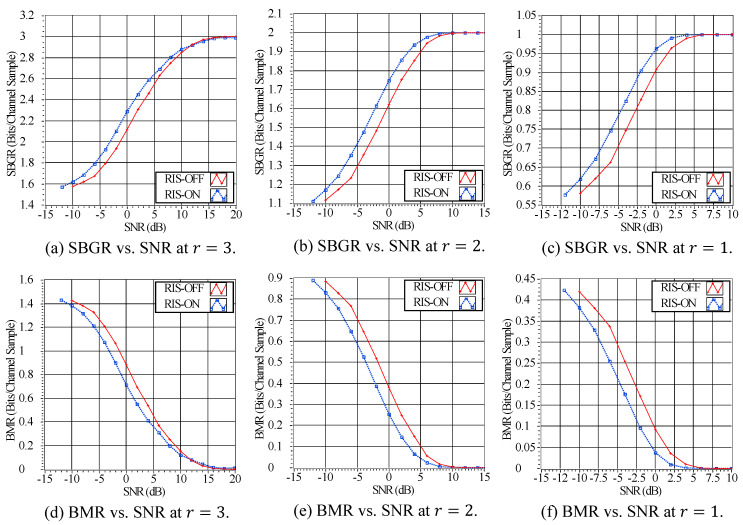
The scheme’s performance of the SBGR and the BMR at different SNRs and r={1,2,3}.

We compare secret key extraction performance under two scenarios: when the RIS is activated with the optimal configuration ($H_{opt}$) and when the RIS is turned off. The performance evaluation is based on the SBGR metric from (7) and the BMR metric from (8), at various SNR values and r={1,2,3}. As presented in Figure 7a–c, the results indicate that the SBGR improves when the RIS is activated. For instance, at an SNR of 0 dB, the SBGR increases from approximately 1.62 bits/sample when the RIS is off to approximately 1.75 bits/sample when the RIS is activated (see Figure 7b). Conversely, the BMR decreases when the RIS is activated as compared to when it is kept off. For instance, at an SNR of 0 dB, the BMR drops from approximately 0.38 bits/sample when the RIS is off to approximately 0.25 bits/sample when the RIS is activated (see Figure 7e). These results demonstrate the efficacy of the RIS in enhancing secret key-extraction performance.

The quantization order, *r*, can be optimized based on the estimated average SNR at the side of Bob, ${\bar{\mathrm{SNR}}}_{H_{opt}}^{Bob}$, corresponding to the optimal configuration $H_{opt}$, where ${\bar{\mathrm{SNR}}}_{H_{opt}}^{Bob}\in {\bar{\mathrm{SNR}}}^{Bob}$ in step (6) from Algorithm 1. The optimization range for an acceptable BMR ≤0.1 bits/sample is presented in Table 2 for scenarios where the RIS is both ON and OFF. It can be inferred that the RIS is more effective in improving the system performance in scenarios with lower SNR values than in higher SNR scenarios. This suggests that the impact of the RIS on the SNR may be limited when the SNR is already high, and other factors, such as fading and shadowing, may have a more dominant impact on the system performance. For the terminals to agree on r=2, the estimated average SNR should be within the range of $5\;\mathrm{dB}\leq {\bar{\mathrm{SNR}}}_{H_{opt}}^{Bob}<12\;\mathrm{dB}$ when the RIS is OFF, and $3\;\mathrm{dB}\leq {\bar{\mathrm{SNR}}}_{H_{opt}}^{Bob}<12\;\mathrm{dB}$ when the RIS is ON. When the estimated average SNR is below the specified range, both terminals can agree on r=1 if ${\bar{\mathrm{SNR}}}_{H_{opt}}^{Bob}<5\;\mathrm{dB}$ when the RIS is OFF, and ${\bar{\mathrm{SNR}}}_{H_{opt}}^{Bob}<3\;\mathrm{dB}$ when the RIS is ON.

Furthermore, the extracted bit streams are rigorously evaluated for statistical defects through the application of the well-established randomness test suite developed by the National Institute of Standards and Technology (NIST) [28]. The results of each test are presented in the form of a p-value for extracted keys with a length of 256 bits, as depicted in Table 3. These values are then compared to the predetermined significance level (0.01) to assess the degree of randomness of the extracted bit streams. It can be observed that the extracted keys exhibit satisfactory randomness properties, as their chaotic characteristics are predominantly determined by the random large integer parameters $n_{i}$ and $m_{i}$ of chaotic mapping operation in (1), selected by the individual users.

### 5.3. Overhead Analysis

This part presents a discussion on the execution time required for Algorithm 1 and the identification of the optimal configuration ($H_{opt}$) to achieve the research objective. The reflecting units of the developed RIS prototype are controlled through positive-intrinsic-negative (PIN) diodes, which switch between two-phase states. The individual control of each unit element allows for operation in the near field and channel estimation. The configuration is generated using a Hadamard codebook in MATLAB, which is transferred over WiFi using a transmission control protocol/internet protocol (TCP/IP) link to a server program running on the Raspberry Pi-3 (Model B). The clock speed of the Raspberry was optimized at 7.8 MHz, with an operational power consumption of 12–15 watts and a beam switching speed of 8 ms. Based on the updating time, the overall running time for 4096 RIS configurations is calculated as 4096×0.008=32.7 s which is acceptable as a prototype RIS with limited performance capabilities. However, this time can be significantly reduced using a high-speed field programmable gate array (FPGA) that operates at a clock speed of up to 500 MHz. Specifically, this would entail updating the control circuits of the PIN diodes to ensure compatibility with the FPGA’s clock speed. This strategy holds the potential to significantly shorten the required running time.

The security robustness of the proposed secret key-extraction scheme depends on the infeasibility of solving the Diffie–Hellman problem through the use of the Chebyshev chaotic mapping operation presented in (1) [5]. This is facilitated by the straightforward multiplication and modular arithmetic operations involved in the calculation of $T_{n_{i}}^{\prime }\left(\theta_{i}\right)$. Hence, the proposed method exhibits significantly reduced computational complexity in comparison to that of the computationally intensive elliptic curve-based Diffie–Hellman key exchanging protocol.

## 6. Conclusions

In this paper, we have investigated the feasibility of employing the RIS to enhance the PHY-layer secret key extraction performance in the presence of DoS attacks. We propose an optimization algorithm that leverages the RIS to boost the signals transmitted by legitimate users while suppressing the interfering signals from malicious adversaries. Furthermore, we have experimentally demonstrated the effectiveness of the proposed RIS-assisted key-extraction method using a 1-bit RIS and two USRPs. Experimental results show that this method enhances the performance of the key extraction, as quantified by two performance metrics, the SBGR and BMR. Specifically, we observed an increase in the SBGR from 1.62 to 1.75 bits/sample when the RIS is turned on and a decrease in the BMR from 0.38 to 0.25 bits/sample when the RIS is enabled at a poor SNR of 0 dB. These findings are particularly significant for future insights into secure and reliable intelligent transportation systems. Additionally, we evaluated the statistical randomness of the extracted keys using the NIST statistical test suite, confirming that the extracted keys are suitable for use as cryptographic keys. In summary, the presented results and analyses offer valuable perspectives on the practical implementation and optimization of the RISs in enhancing the security and functionality of the PHY-layer secret key extraction for poor SNR and NLoS scenarios. Our future work will examine the possibility of employing the extracted key for designing an efficient message-authentication scheme for VANET applications, exploring the practicality of implementing it in a realistic vehicular channel.

## Figures and Tables

**Table 1:** List of notations.

Symbol	Definition
$\theta_{i}$	The generator of the cyclic group G for the *i*th subcarrier of the OFDM symbol
$n_{i},m_{i}$	The private integer numbers at the sides of Alice and Bob, respectively
Δt	The transmission time interval between two subsequent OFDM symbols
$|h_{i}|,\xi_{i}$	The wireless-channel amplitude and phase responses, respectively
${\hat{T}}_{m_{i}}^{\prime }\left(\theta_{i}\right),{\hat{T}}_{n_{i}}^{\prime }\left(\theta_{i}\right)$	The equalized phase estimates at the sides of Alice and Bob, respectively
$M^{-1}$	The Gray code mapping operation that converts final estimates into bit streams
*r*	The order of the generator $\theta_{i}$ and the mapping operation $M^{-1}$
ϕ(x),erf(z)	The cumulative distribution function and the error function
$P_{e}$	The probability of error in the extracted key between two parties
*I*	The secret key capacity
HD	The Hadamard codebook used for optimizing the RIS configuration
${\bar{\mathrm{SNR}}}_{i}^{Bob},{\bar{\mathrm{SNR}}}_{i}^{Eve}$	The average SNR of the signals transmitted from Bob and Eve, respectively
$H_{opt}$	The optimal configuration for the RIS’s reflecting units

**Table 2:** The optimized SNRs for r={1,2,3}, with/without the RIS, and the BMR ≤0.1 bits/sample.

Quantization Order	RIS Status (ON/OFF)	
	RIS-OFF	RIS-ON
r=3	SNR ≥12dB	SNR ≥12dB
r=2	5dB≤SNR<12dB	3dB≤SNR<12dB
r=1	SNR ≤5dB	SNR ≤3dB

**Table 3:** Statistical randomness analysis of the extracted keys.

NIST Statistical Test Suite (256 bits)	*p*-Value
Key Entropy	0.299629
Monobit Test	0.59766
Long Runs Test	0.485934
Block Frequency Test	0.486333
Maurer Universal Statistical Test	0.156093
Overlapping Template Matchings Test	0.486245
Discrete Fourier Transform (Spectral) Test	0.507344

## Data Availability

Data will be made available on request.
